# Isobaric tags for relative and absolute quantification-based proteomic analysis of host-pathogen protein interactions in the midgut of *Aedes albopictus* during dengue virus infection

**DOI:** 10.3389/fmicb.2022.990978

**Published:** 2022-09-14

**Authors:** Jiatian Wang, Peiyang Fan, Yong Wei, Jiaqi Wang, Weihao Zou, Guofa Zhou, Daibin Zhong, Xueli Zheng

**Affiliations:** ^1^Department of Pathogen Biology, School of Public Health, Southern Medical University, Guangzhou, China; ^2^Program in Public Health, College of Health Sciences, University of California, Irvine, Irvine, CA, United States

**Keywords:** *Ae. albopictus*, DENV, iTRAQ, PC protein, UCH protein

## Abstract

Aedes albopictus (Ae. albopictus), an important vector of dengue virus (DENV), is distributed worldwide. Identifying host proteins involved in flavivirus replication in *Ae. albopictus* and determining their natural antiviral mechanisms are critical to control virus transmission. Revealing the key proteins related to virus replication and exploring the host-pathogen interaction are of great significance in finding new pathways of the natural immune response in *Ae. albopictus*. Isobaric tags for relative and absolute quantification (iTRAQ) was used to perform a comparative proteomic analysis between the midgut of *Ae. albopictus* infected with DENV and the control. 3,419 proteins were detected, of which 162 were ≥ 1.2-fold differentially upregulated or ≤ 0.8-fold differentially downregulated (*p* < 0.05) during DENV infections. Differentially expressed proteins (DEPs) were mainly enriched in ubiquitin ligase complex, structural constituent of cuticle, carbohydrate metabolism, and lipid metabolism pathways. We found that one of the DEPs, a putative pupal cuticle (PC) protein could inhibit the replication of DENV and interact with the DENV-E protein. In addition, the result of immunofluorescence (IF) test showed that there was co-localization between ubiquitin carboxyl-terminal hydrolase (UCH) protein and the DENV-E protein, and virus infection reduced the level of this protein. iTRAQ-based proteomic analysis of the *Ae. albopictus* midgut identified dengue infection-induced upregulated and downregulated proteins. The interaction between the PC and UCH proteins in the midgut of *Ae. albopictus* might exert a natural antiviral mechanism in mosquito.

## Introduction

*Aedes albopictus* is one of the principal vectors for the transmission of dengue virus (DENV) and has a worldwide distribution (Goenaga et al., 2020). DENV is a globally important mosquito-borne flavivirus that can lead to a widespread human disease and death. From 1990 to 2013, the number of dengue fever cases doubled every decade, and reached an estimated 390 million DENV infections in 2010, of which 96 million resulted in symptomatic disease (Bhatt et al., 2013), with an estimated 13,600 deaths each year (Shepard et al., 2016). Moreover, DENV is also considered to be the second leading cause of acute febrile diseases in travelers (Freedman et al., 2006; Guzman et al., 2010). However, currently, there is no specific treatment for dengue fever, and the vaccine requires improvement because of safety concerns due to the potential induction of severe symptoms due to antibody-dependent enhancement (Robinson and Durbin, 2017). In previous dengue fever epidemics in China, *Ae. albopictus* was the main transmission vector; thus, effective restriction of DENV replication in mosquitoes is vital to control the spread of the virus. DENV, a member of the flavivirus family, contains three structural proteins [capsid (C), membrane protein (M), and envelope glycoprotein (E)] and seven non-structural proteins (NS1, NS2a, NS2b, NS3, NS4a, NS4b, and NS5) (Mukhopadhyay et al., 2005), usually replicate within a mosquito vector for 7–10 days and then the vector can transmit the virus to humans through bites (Blair et al., 2000; Mackenzie et al., 2004). When *Ae. albopictus* is infected with DENV, the virus must first cross the midgut infection barrier, establish infection in the midgut, and replicate in the midgut epithelial cells. During the replication process, flaviviruses manipulate the host cell system to promote its replication, and the host cell activates an antiviral response (Zhang et al., 2013). The virus must then cross the mosquito’s midgut escape barrier to infect other tissues. As the first tissue infected by DENV, the midgut plays an important role in the innate immune response of *Ae. albopictus* in the early stage of virus infection (Wang et al., 2018). The identification of host proteins of midgut involved in flavivirus replication might lead to the discovery of antiviral targets (Ma-Lauer et al., 2012). Advances in mosquito genomics and molecular biology have greatly facilitated the study of virus-mosquito interactions and molecular-level antiviral mechanisms (Nene et al., 2007; Arensburger et al., 2010; Neafsey et al., 2015). Innate immunity-related genes have been the focus of many of these studies, and the Toll pathway, interferon regulation, and signal transducer and activator of transcription (STAT) pathways have emerged as important anti-flaviviral mechanisms in the mosquito (Cheng et al., 2016). Previous study found that NS5 protein of DENV binds to STAT2, leading to ubiquitination of STAT2, which reduces the type I interferon-mediated immune response and then increases the replication of DENV (Mazzon et al., 2009). NS5 of Zika virus (ZIKV) promoted the degradation of STAT2 (Grant et al., 2016; Kumar et al., 2016), while NS2B3 can degrade Janus Kinase 1 (JAK1) (Wu et al., 2017), thus inhibiting JAK-STAT signaling and coincidentally impairing downstream interferon-stimulated gene (ISG) expression.

However, little research has focused on the protein level changes in the midgut of *Ae. albopictus* infected with DENV. Most previous studies used the virus overlay binding protein assay (VOBPA) method or two-dimensional electrophoresis to analyze protein levels, and protein identification was limited to the molecular weight, or only focused on the analysis at the cell level (Ghosh et al., 2019). Isobaric tags for relative and absolute quantification (iTRAQ), as a highly advanced proteomic platform, can identify differentially regulated proteins more accurately and provide reliable quantitative information. This method labels protein samples with unique isotopes to enable robust comparisons of different samples in a mixture and to study changes in protein levels under different experimental conditions (De Mandal et al., 2020; Zhang et al., 2021). iTRAQ has been applied successfully to screen differentially expressed proteins (DEPs) after pathogen infection in mosquitos to understand host immune defense mechanisms (Baldridge et al., 2017; Xin et al., 2017). The present study aimed to carry out an iTRAQ-based quantitative proteomic analysis of DENV-infected *Ae. albopictus* to identify changes in protein levels after DENV infection, investigate host proteins involved in the DENV infection process, explore the interaction between the proteins and virus, and provide the basis for the development of new mosquito vector controls tools.

## Materials and methods

### Cell culture and virus enrichment

C6/36 cells, derived from *Ae. albopictus*, were used for DENV-2 (GenBank: AF038403.1) propagation. The cells were cultured in minimal essential medium (RPMI 1640; GIBCO, Invitrogen, Waltham, MA, United States) supplemented with 10% fetal bovine serum (FBS) and maintained at 28 °C. Cells grown in a 25 cm^3^ culture flask were inoculated with DENV-2 at a multiplicity of infection (MOI) of 1. After gentle shaking for 15 min, the culture flask was incubated at 37°C in 5% CO_2_ for 2 days until obvious cytopathic effects were observed. The supernatant was harvested after centrifugation at 800 × *g* for 5 min, separated into 1 mL aliquots, and frozen at −80°c.

BHK-21 cells, derived from baby Syrian hamster kidney, were used for the immunofluorescence (IF) experiment. COS7 cells, derived from African green monkey kidney, were used for the Immunoprecipitation (IP) Assay. The cells were grown at 37°C and 5% CO_2_ in Dulbecco’s modified Eagle’s medium (DMEM) supplemented with 10% FBS and 1% penicillin-streptomycin.

### Experimental infection of mosquitoes with dengue virus-2 virus

*Ae. albopictus* used in this study was the Foshan strain, which was collected from Fushan, Guangdong Province, China, in 1981. Mosquitoes were kept in a environmental chamber in the insectary with a temperature of 28°C, humidity 80%, and a 16 h:8 h light-dark photoperiod. Females 4–6 days were used and starved for 12 h before experiments. For the experimental group: The DENV-2 supernatant was collected and mixed with defibrinated sheep blood at a ratio of 2:1. For the control group: the cell culture supernatant and sterile defibrinated sheep blood were mixed 2:1 to prepare a blood meal. The mixtures were stored at 37°C for 30 min, and fed the starved female mosquitoes for 1 h. The fully blood-fed female mosquitoes were picked out and raised in a new container with 10% glucose. The infectivity experiment was repeated three times.

### qRT-PCR analysis

The RNA from the *Ae. albopictus* midguts were extracted by TRIzol reagent (AG RNAex Pro RNA, Accurate Biology, Hunan, China) according to the manufacturer’s instructions and reverse transcribed to cDNA using a GoScript Reverse Transcriptase System (Promega, Madison, WI, United States). A Power Green qPCR Mix kit (SYBR Green 1) (GDSBio, Guangzhou, China) was used to quantify the cDNA to detect the viral load and related gene expression in the midgut of *Ae. albopictus. Rps7* (encoding ribosomal protein S7) was used as an internal reference gene. The primers used are shown in Supplementary Table 1.

### Protein extraction from mosquito midguts

When the infection rate in the experimental group reached 80%, the viral load was higher than 10^7^ *copies* per midgut, and the control group was not infected with virus, the infections were considered effective. On day 14 post-infection, the midguts of the remaining samples were dissected, and 80 midguts were placed into an EP tube containing 50 mL pre-cooled phosphate-buffered saline (PBS) for each sample. After collection, the samples were centrifuged at 12,000 × *g* and 4°C for 2 min, the supernatant was discarded. The above steps were repeated, the protein pellet was washed three times with PBS and centrifuged, and the pellet was stored at −80°C before protein extraction. When all the midgut samples were collected, 1 mL of lysis buffer (Beyotime, Shanghai, China) was added the samples and the samples were ground with a homogenizer for 1–2 min. The samples were centrifuged at 4°C, 13,000 × *g* for 10 min, and the protein in the supernatant was precipitated by adding three volumes of acetone. The protein pellet was collected by centrifugation and the protein concentration was measured using a modified Bradford Protein Assay Kit (Thermo Fisher Scientific, Waltham, MA, United States) according to the manufacturer’s instructions.

### Trypsin digestion and isobaric tags for relative and absolute quantification labeling

For each sample, 100 μg of protein was used for digestion and iTRAQ labeling. Firstly, 10 mM DTT was added to the protein sample and samples were incubated at 37°C for 45 min. The proteins were then alkylated by incubation in 25 mM iodoacetamide for 55 min at room temperature in the dark. The urea concentration of the samples was diluted to less than 2 M using 100 mM Tetraethylammonium bromide (TEAB) and then proteins were digested using Sequencing Grade Modified Trypsin (first trypsin digestion: protein:trypsin = 50:1 mass ratio at 37°C overnight; second trypsin digestion: protein:trypsin = 100:1 for 4 h). After trypsin digestion, the peptides were desalted on a Strata X SPE column, dried *in vacuo* and reconstituted in 20 μL of 500 mM TEAB. The peptides were then labeled using an 8-plex iTRAQ kit according to the manufacturer’s protocol. The peptides were dried and reconstituted with high performance liquid chromatography (HPLC) solution A (2% acetonitrile (ACN), pH 10) and fractionated using high pH reverse-phase HPLC using a Waters Bridge Peptide BEH C18 column (130 Å, 3.5 μm, 4.6*250 mm; Milford, MA, United States). Briefly, the peptides were first separated into 72 fractions in 88 min at a rate of 0.5 mL/min at pH 10 with a gradient of 2–98% ACN. Then, the 72 tubes of the mixture for the liquid phase separation of the peptides were combined into 18 fractions and were dried by vacuum centrifugation and desalted using ZipTip C18 tips according to the manufacturer’s instructions (Merck Millipore, Billerica, MA, United States; ZiptTip Pipette Tips, 10 μL). Finally, the samples were dried under a vacuum and maintained at −20°C until mass spectrometry analysis.

### High-resolution liquid chromatography with tandem mass spectrometry analysis

The experiment was carried out using Nano-LC 1000 LC-MS / MS with a proxeon easy NLC 1000 coupled with a mass spectrometer (Thermo Fisher Q exactive, United States) for fraction separation. Trypsinized fractions were loaded into buffer A (0.1% formic acid) connected to the reverse phase capture column of the C18 reverse phase analysis column, and separated using a linear gradient of buffer B (84% ACN and 0.1% formic acid). The eluent was analyzed by tandem mass spectrometry (MS/MS) in Q Exactive after spraying *via* a nanoelectrospray source at 2.0 kV electrospray voltage. MS data were obtained using a data-dependent mode that dynamically selected the 20 most intense precursor ions present in the survey scan (350–18,000 M/Z) for higher-energy C-trap dissociation fragmentation. In the Orbitrap mass analyzer, survey scans were acquired at a resolution of 70,000 (full width at half maxima, FWHM) and ion fragments were detected at a resolution of 17,000 (FWHM). The collision energy was 28% in the MS survey scan with 10.0 s of dynamic exclusion (De Mandal et al., 2020).

### Data processing and bioinformatic analysis

The MS/MS raw data were searched against the Uniprot database (ST7160_*AedesAlbopictus*_25914_20201215) by using Sequest software integration in Proteome Discoverer (version 1.3, Thermo Scientific, Waltham, MA, United States), with the following parameter settings: (a) Enzyme: Typsin/P; (b) Missed Cleavages: 2; (c) Fixed Modification: Caramidomethyl (C); (d) Variable Modifications: Acetyl (N-term), Oxidation (M), iTRAQ (K,Y), iTRAQ (N-term);(e) Peptide Tolerance: 20 ppm; (f) MS/MS Tolerance: 0.05 Da; (g) Use Target-Decoy, and Peptide false discovery rate (FDR) < 0.01. The functional annotations of all the DEPs were performed using Blast2go_v2.5 against the NCBI database. Gene Ontology (GO),^1^ Cluster of Orthologous Groups of Proteins (COG),^2^ and Kyoto Encyclopedia of Genes and Genomes (KEGG)^3^ were used to analysis the functions of the DEPs.

### Protein production and plasmid constructs

The coding sequences of A0A023EEV6, which encodes a putative pupal cuticle protein (PC, GenBank: LOC109414002) and A0A023EXQ2, which encodes a ubiquitin carboxyl-terminal hydrolase (UCH, GenBank: LOC115254107), were amplified from mosquito C6/36 cell cDNA using RT-PCR. Hemagglutinin (HA)-tagged versions of these two proteins were made by cloning the two amplified genes into the pAC5.1b-myc and pcDNA3.1 (+) expression vectors, respectively, and then inducing protein expression.

### Transfection assays and infection assays

C6/36 cells were transfected with the plasmids constructed from the pAC5.1b-myc expression vector, BHK-21 and COS7 cells were transfected with pcDNA3.1 (+) vector. For plasmid DNA transfection, cells at 80% confluence were treated with Lipofectamine 3000 transfection reagent (Thermo Fisher Scientific, United States) as recommended by the manufacturer. At 6 h post-transfection, the medium was replaced with fresh complete growth medium. The cells were infected with DENV virus at 24–48 h post-transfection, and proteins were collected or other follow-up experiments were performed 24 h after virus infection.

### Western blotting analysis

The same amount (about 30 μg) of extracted protein samples were separated using sodium dodecyl sulfate polyacrylamide gel electrophoresis (SDS-PAGE) and transferred to a polyvinylidene fluoride (PVDF) membrane (Millipore). After blocking with Tris-buffered saline with Tween 20 (TBST) containing 5% bovine serum albumin (BSA), the membranes were incubated with primary antibodies followed by incubation with the corresponding horseradish peroxidase-conjugated secondary antibodies. The immunoreactive proteins on the membrane were detected by using Clarity Western ECL Substrate (Bio-Rad, Hercules, CA, United States) and photographed using a ChemiDoc Touch Imaging System (Bio-Rad). The primary antibodies comprised a mouse anti-DENV-E monoclonal antibody (1:5,000; Invitrogen, Waltham, MA, United States), a mouse anti-glyceraldehyde-3-phosphate dehydrogenase (GAPDH) monoclonal antibody (1:2,000; PTM Biolabs, Hangzhou, China), and a rabbit anti-HA monoclonal antibody (1:2,000; PTM Biolabs). The secondary anti-mouse and anti-rabbit horseradish peroxidase-conjugated antibodies (Abcam, Cambridge, MA, United States) were used at a dilution of 1:5,000.

### Immunofluorescence assay

BHK-21 cells were used for IF analysis. HA-tagged plasmids were transfected into the cells. At 24 h after transfection, DENV virus was infected into the cells. At 24 h post-infection, we fixed the cells with 4% paraformaldehyde, permeabilized them using 0.1% Triton X-100, and blocked them using 1% FBS (Biofroxx, Einhausen, Germany). The cells were then incubated with primary antibodies overnight at 4°C and then stained with secondary antibodies for 1 h at room temperature. Cells mounted on coverslips were imaged using a confocal microscope (ELWD 0.3T1-SNCP, Nikon, Japan). For PC protein detection, a yellow fluorescent protein (YFP)-PC fusion protein was expressed, thus we only need to incubate the cells with anti-DENV-E antibodies (1:5,000, Invitrogen), followed by incubation with the fluorescent secondary antibody (Alexa Fluor^®^ 594, Invitrogen). For UCH, the cells were incubated with anti-DENV-E antibodies and anti-HA-tag [phycoerythrin (PE)] antibodies (PTM Biolabs) as primary antibodies and incubated with the fluorescent secondary antibody (Alexa Fluor 488, Invitrogen).

### Immunoprecipitation assay

COS-7 cells overexpressing PC were prepared as detailed above. When the cells in T25 (25 cm^3^ Flask, Corning, NY, United States) were about 80% confluent, 3 μg of the pcDNA3.1 (+)-PC plasmid were transfected into the COS-7 cells for the experimental group, using Lipofectamine 3000 transfection reagent. At 24 h post-transfection, the cells were infected with DENV. 24 h later, cell extracts were prepared and incubated with the primary antibody (anti-HA rabbit monoclonal antibody, PTM Biolabs, China) with gentle rotation for 2 h at 4°C. Protein A-Resin (Transgen, Beijing, China) was then added to the reaction and incubated overnight at 4°C. The immunoprecipitates were washed four times with PBS buffer and then eluted by boiling with SDS-PAGE loading buffer (Transgen). The eluates were analyzed by western blotting as described above.

## Results

### Virus infection in the midgut tissue of *Aedes albopictus*

To ensure that the viral load of the midgut samples in the experimental group was at a high level, and the control group was not infected with virus, we examined viral loads using qRT-PCR from 10 *Ae. albopictus* midguts in each group on day 12 post-infection. The experiment was repeated three times, and the results showed that the control group was not infected with DENV virus and the infection rates of the experimental group were greater than 80% (Supplementary Table 2).

### Identification of proteins altered by dengue virus infection

**FIGURE 1:**
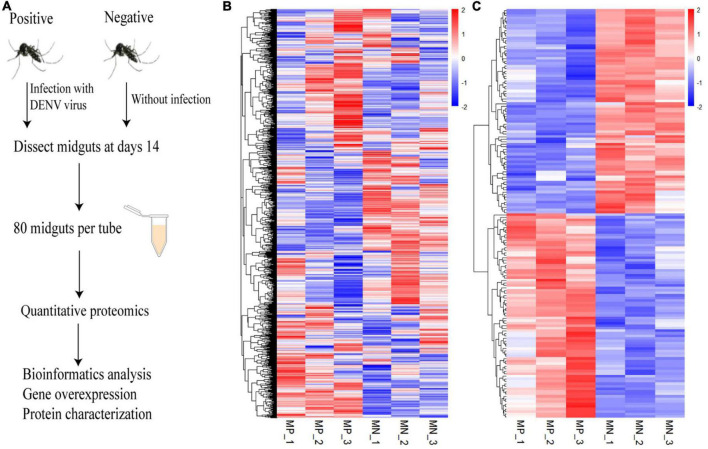
Quantitative proteomics of the *Ae. albopictus* midgut during DENV infection. **(A)** Schematic diagram of the experimental procedure. *Ae. albopictus* mosquitoes were infected with DENV. Quantitative proteomics analysis was carried out at 14 days post infection. **(B)** The levels of 3,419 proteins between the infected and uninfected *Ae. albopictus* groups were determined by three repeated experiments. **(C)** Heat map of differential expressed proteins with a ratio of more than 1.2 times. Each column in the diagram represents a sample and each row represents a protein, and red represents a high levels and blue represents a low level. MP means mosquito-positive group, MN means mosquito-negative group.

On day 14 post infection, midguts were dissected from the mosquitoes and the samples were analyzed by using iTRAQ method to determine alterations in protein levels between the mock and infected groups. The experiment schematic is shown in Figure 1A. A total of 3,419 proteins were identified in this experiment, the expression levels of all identified proteins were analyzed and further visualized. We screened the protein level between the experimental and control groups, and used the R software 64 4.1.1 to draw the heatmap for the three biological repetitions, and then clustered each protein in the horizontal direction and each sample in the vertical direction (Figure 1B). Furthermore, we screened the DEPs whose level showed > 1.2 times difference between the two groups and displayed them in the heat map (Figure 1C). According to the difference criterion (*P* < 0.05 and Ratio < 1.2), 162 DEPs were screened out, including 81 upregulated proteins and 81 downregulated proteins (Supplementary Figure 1). The details of the DEPs are shown in Supplementary Table 3. The repeatability analysis showed that the three biological repetitions were highly correlated, indicating that the experimental results were reliable (Supplementary Figure 2). Networks of interactions of DEPs were shown in Supplementary Figure 3.

### Functional classification and bioinformatic analyses of differentially expressed proteins

**FIGURE 2:**
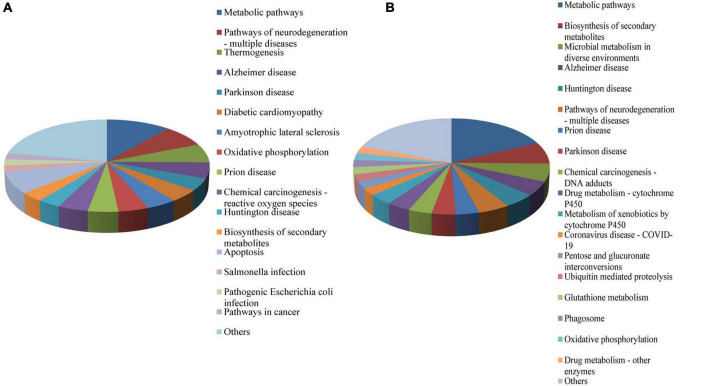
KEGG functional classification of the DEPS in the midgut of *Ae. albopictus* identified by iTRAQ analysis. **(A)** KEGG functional classification of upregulated proteins; **(B)** KEGG functional classification of downregulated proteins. DEP, differential expressed protein; KEGG, Kyoto Encyclopedia of Genes and genomes.

To understand the biological functions of the DEPs, KEGG ontology assignments were used to classify their functional annotations. KEGG pathways associated with the upregulated DEPs included Metabolic pathways (11%), Pathways of neurodegeneration—multiple diseases (7.7%), Apoptosis (7.7%), Thermogenesis (6.6%), Alzheimer disease (5.5%), Oxidative phosphorylation (4.4%), and Prion disease (4.4%) (Figure 2A). KEGG pathways associated with the downregulated DEPs included Metabolic pathways (18.1%), Biosynthesis of secondary metabolites (7.2%), Microbial metabolism in diverse environments (6.0%), Pathways of neurodegeneration—multiple diseases (4.8%), Prion disease (3.6%), Drug metabolism—cytochrome P450 (3.6%), Metabolism of xenobiotics by cytochrome P450 (3.6%), Coronavirus disease—COVID-19 (2.4%), and Ubiquitin mediated proteolysis (2.4%) (Figure 2B).

**FIGURE 3:**
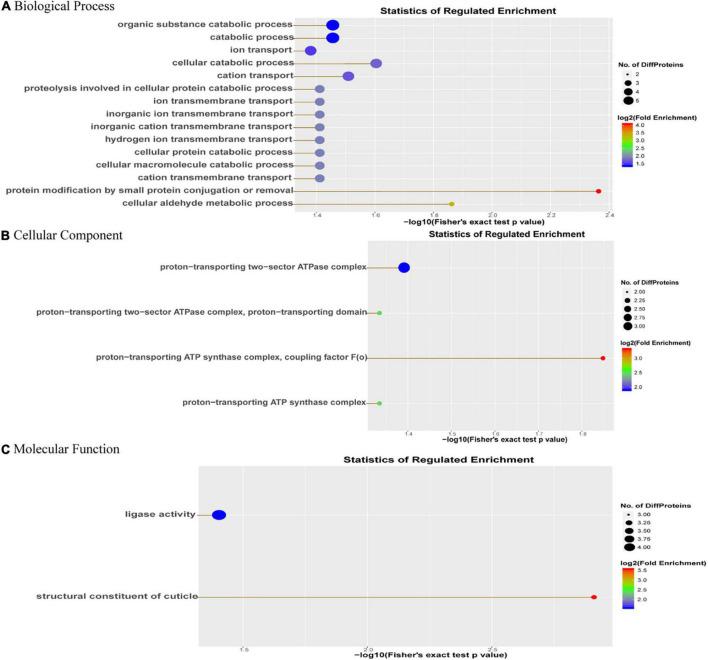
GO enrichment analysis of DEPs. The abscissa is the **(A)** GO-biological process enrichment; **(B)** GO-cellular component enrichment; and **(C)** GO-molecular function enrichment. The abscissa is the *p*-value of log10 conversion. The larger the value, the higher the significance of the enrichment in this category. The ordinate represents the name of the enrichment category, and the larger the circle, the more the number of differential proteins in this category. The color of the circle represents the fold enrichment of the protein, and the redder the color, the greater the enrichment.

In order to further understand the biological pathways of DEPs, we carried out GO enrichment analysis of DEPs from three aspects: biological process (BP), molecular function (MF) and cellular component (CC). The results showed that in terms of GO-CC, the DEPs were mainly enriched in proton-transporting ATPase synthase complex, coupling factor; in terms of GO-BP, the DEPs were significantly enriched in protein modification by small protein conjugation or removal; in terms of GO-MF, the DEPs were significantly enriched in the structural constituents of the cuticle (Figure 3).

### Verification of the differentially expressed proteins with RT-qPCR

**FIGURE 4:**
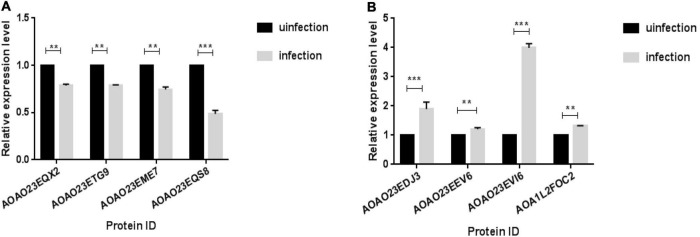
qPCR validation of transcript levels between infection and infection groups. Midguts were dissected from mosquitoes and RNA was extracted on day 14th after the mosquito infected with DENV, qPCR was used to verify the expression levels of DEPs between the infected group and the uninfected group. **(A)** The down-regulation proteins of infection group. **(B)** The up-regulation proteins of infection group (**P < 0.01, ***P < 0.001).

RT-qPCR was used to detect the transcription levels of four up-regulated proteins (A0A023EDJ3-Putative acp65aa; A0A023EEV6-Putative pupal cuticle protein; A0A023EVI6-Putative e3 ubiquitin ligase cullin 2 component; A0A1L2F0C2-Caspase 7) and four down-regulated proteins (A0A023EQX2-Ubiquitin carboxyl-terminal hydrolase; A0A023ETG9-Tubulin beta chain; A0A023EME7-Putative glutathione s-transferase; A0A023EQS8-Putative aldo/keto reductase family) from the significantly enriched DEPs associated with structural constituents of the cuticle, pathways of neurodegeneration—multiple diseases, apoptosis and Metabolic. Results illustrate that the expression level of these DEPs were significantly differently expressed (*P*< 0.01) in the infectious group and control group (Figure 4). Moreover, similar trends were observed in gene expression at both transcriptomics and proteomics levels among the infectious and control groups of the *Ae. albopictus*’ midgut.

### Pupal cuticle protein inhibits dengue virus-2 infection

**FIGURE 5:**
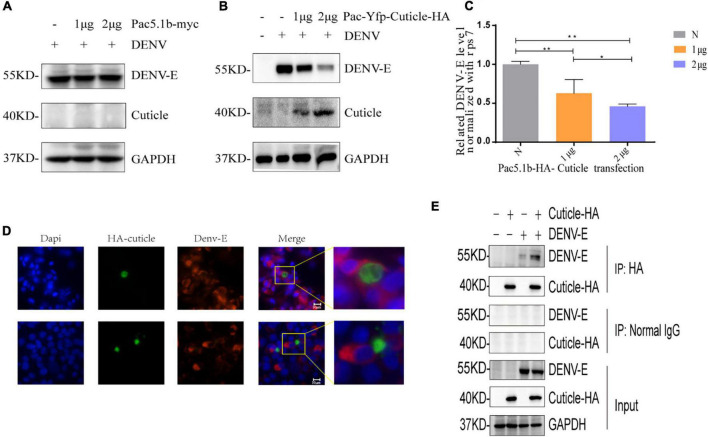
PC protein inhibits the expression of DENV-E protein. **(A)** Empty vector were transfected into C6/36 cells, and cells were inoculated with DENV (MOI = 1) 48 h after transfection, lysates of C6/36 cells at 24 hpi were detected by SDS-PAGE and WB using the antibodies as indicated. **(B,C)** 0, 1, 2 μgpAC5.1b-myc-YFP-Cuticle-HA was transfected into C6/36 cells, respectively, and DENV (MOI = 1) was infected 48 h after transfection. The cells’ RNA and protein were extracted from the cells at 24 hpi and detected by qRT-PCR and WB as indicated, respectively. **(D)** BHK-21 cells were transfected with pcDNA3.1(+)-YFP-Cuticle-HA plasmids and infected with DENV (MOI = 1) at 24 h after transfection. Cells were fixed at 24 hpi, and incubated with a DENV-E protein monoclonal antibody and the corresponding fluorescent secondary antibody. A YFP-PC-HA fusion protein was expressed to detect PC protein (green), localization of DENV-E was detected with antibodies to fluorescent secondary antibody (red), nuclei were stained with DAPI (blue). The upper and lower images in each column are of two fields of view in the same slide. **(A,E)** COS7 cells were transfected with pcDNA3.1 (+)-YFP-EEV6-HA plasmid and infected with DENV (MOI = 1) at 24 h after transfection, and proteins were extracted at 24 hpi for WB and Co-ip analysis. All experiments were performed at least three times, *denotes a *P-*value < 0.005, **denotes a *P-*value < 0.001.

We hypothesized that interaction of the mosquito proteins with DENV might inhibit virus replication. It has been previously reported that a pupal cuticle protein can bind West Nile envelope protein (WNE) in mice to inhibit the infection *in vitro* (Colpitts et al., 2011). Besides, previous knowledge indicated that cuticular proteins of insect may facilitate virus entry at the gut level, and assist virus particles in the hemolymph (Liu et al., 2015). To confirm that the increase of cuticle protein can inhibit DENV replication, recombinant protein was generated from one of the upregulated proteins, A0A023EEV6, which codes for a putative pupal cuticle protein (PC). Firstly, pAC5.1b-myc empty plasmid vector was transfected into C6/36 cells to prove that the transfection of the vector have no effect on the replication of DENV (Figure 5A). Then, we transfected C6/36 cells with PAC5.1b-YFP-Cuticle-HA and infected them with DENV and found that with the increasing concentration of transfected PC protein plasmid, the expression of dengue-E protein showed a decreasing trend at the protein level (Figure 5B), which was consistent with the results at the transcriptional level (Figure 5C). The result showed that PC could inhibit the expression of DENV-E in a dose-dependent manner.

In addition, to confirm how PC protein inhibit the expression of DENV, we transfected pcDNA3.1 (+)-YFP-cuticle-HA into BHK-21 cells for an IF assay. Interestingly, the results showed that the expression of DENV-E protein was extremely weak and almost undetectable in cells overexpressing PC, while there was no expression of PC in cells with high levels of DENV-E protein (Figure 5D), indicating that PC might affect the invasion of DENV into host cells. In the results of co-immunoprecipitation in COS7 cells, cuticle-HA protein and DENV-E protein could be detected in the immunoprecipitated proteins of the experimental group, while the proteins were not detected in the negative control group, indicating that there is an interaction between PC and DENV-E protein (Figure 5E).

### Dengue virus-2 infection results in decreased expression of ubiquitin hydrolase

**FIGURE 6:**
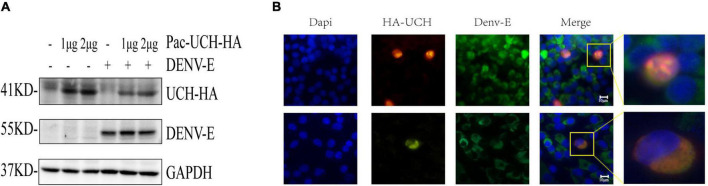
DENV infection results in degradation of UCH. **(A)** pAC5.1b-myc-UCH-HA plasmids were transfected into C6/36 cells with a gradient of 0, 1, and 2 μg, and DENV (MOI = 1) was infected 48 h after transfection. Cellular proteins were extracted at 24 hpi. **(B)** 1.5 μg of pcDNA3.1 (+)-UCH-HA plasmids were transfected into BHK-21 cells, and 24 h after transfection, dengue virus (MOI = 1) was infected. The cells were fixed at 24 hpi for immunofluorescence assay. Localization of DENV-E and HA-UCH were detected by co-immunostaining with antibodies to fluorescent secondary antibody (green) and HA tag (red). Nuclei were stained with DAPI (blue). The upper and lower images in each column are of two fields of view in the same slide.

We hypothesized that DENV infection would activate the deubiquitination pathway in the host, and trigger the host’s innate immune response; therefore, a downregulated protein, ubiquitin carboxyl-terminal hydrolase protein (UCH, encoded by A0A023EQX2), was selected to verify the hypothesis. As shown in Figure 6A, the level of UCH was reduced after infection with DENV, suggesting that DENV could degrade UCH or inhibit its gene expression. We next transfected pcDNA3.1 (+)-UCH-HA into BHK-21 cells for an IF assay. The results showed that UCH co-localized with the DENV-E protein (Figure 6B), suggesting that there might be an interaction between the two proteins; however, the mechanism is unclear and requires further verification.

## Discussion

The innate immune system of mosquitoes consists of a variety of different responses, including phagocytosis, nodulation, encapsulation, as well as signaling pathways, such as Janus kinase-signal transducer and activator of transcription (JAK-STAT) and the Toll and immune deficiency (Imd) pathways (Tomari and Zamore, 2006; Tsai et al., 2009; Lee et al., 2012; Huang et al., 2013; Lindsey et al., 2021). Understanding the host’s innate immune system is important to control mosquito-borne virus transmission. The midgut acts as the first natural barrier in *Ae. albopictus* against pathogen invasion. Uncover the DEPs in response to infection with DENV in the midgut of *Ae. albopictus* might help to identify pathways representing both mosquito innate immunity and anti-viral mechanisms. However, there have been few studies on the proteomics of *Ae. albopictus*. To the best of our knowledge, this is the first iTRAQ study of the molecular mechanisms of the midgut defense system of *Ae. albopictus* against DENV. In our study, 3,419 proteins were identified, among which 162 were differentially regulated as a result of DENV infection in the midgut of *Ae. albopictus.* To better understand the response of *Ae. albopictus* to DENV, we classified the DEPs in terms of protein function after virus infection, and found that the DEPs were mainly concentrated in cell metabolism, apoptosis, and the composition of keratin, and post-translational modifications.

KEGG pathways recognized in upregulated DEPs were mainly associated with Metabolic pathways, Pathways of neurodegeneration—multiple diseases, Apoptosis pathways. KEGG pathways recognized in the downregulated DEPs were mainly associated with Metabolic pathways, Biosynthesis of secondary metabolites, Microbial metabolism in diverse environments. GO ontology analysis was carried out for identified the DEPs between the infected and mock groups of *Ae. albopictus*, with enrichment analysis being performed on the three aspects of cell component (CC), biological process (BP), and molecular function (MF). In terms of GO-BP, the DEPs were mainly concentrated in protein modification by small protein conjugation or removal functions; in the aspect of GO-CC, the DEPs were mainly in proton-transporting ATPase synthase complex, coupling factor; while in GO-MF, the enrichment of DEPs was more significant in structural constituent of the cuticle. These results indicated that DENV likely alters host gene expression through both direct and indirect mechanisms during infection of the mosquito which is similar to other researches’ results (Koh et al., 2018; Tsujimoto et al., 2018). Revealing host factors (proteins) that are regulated during viral infection of mosquito might be helpful for identifying conserved protein families and pathways representing both mosquito anti-viral mechanisms and requirements for viral life cycles. Our analysis highlights that many mosquito proteins are associated with DENV infection in mosquitoes.

Oxidative metabolism is very important during mosquito infection with DENV. Previous studies have described that DENV infection can regulate the mitochondrial transmembrane potential and induced changes in mitochondrial respiration in C6/36 cells and increase the production of reactive oxygen species (ROS) oxygen (Santana-Román and Maycotte, 2021). Cells can respond to these changes by increasing the activities of enzymes such as glutathione S-transferase (GST) which comprise a group of multifunctional proteins that are widely distributed in eukaryotes and other organisms (Allocati et al., 2018). In our study, A0A023EME7 was down-regulated in the midgut of *Ae. albopictus* in the infected group, the reason why the expression level of GST did not increase after infection with the virus, but decreased instead, may be that the level of ROS need to be increased in the immune response of mosquitoes. Olagnier et al. (2014) have reported that in primary human monocyte-derived dendritic cells (Mo-DC), increased ROS led to the activation of bystander Mo-DC which up-regulated maturation/activation markers and were less susceptible to viral replication. However, a decrease in ROS levels dampened the innate immune responses to DENV infection and facilitated DENV replication. In addition, previous studies reported that a decrease in GST activity was also observed in Bombyx mori infected with the Bombyx mori nuclear polyhedrosis virus or densonu cleosis virus after an initial increase on the first day post-infection, followed by a decreasing trend afterward (Gui et al., 2009). When tick cell line ISE6 cells were infected with langat virus, up-regulation of the IsGST1 gene was observed on the second day of infection, while down-regulated of the IsGST1 gene was observed on days 3 and 4 after infection (Hernandez et al., 2021).

Studies have reported increased expression and activation of multiple components of the apoptotic cascade in the *Ae. aegypti* midgut after viral infection, suggesting that apoptosis is part of the anti-viral defense (O’Neill et al., 2015; Clem, 2016). The upregulation of apoptosis-related proteins, such as A0A1L2F0C2, A0A182GF96 (Uncharacterized protein), and A0A023ET05 (High temperature requirement protein A2), indicated that apoptosis might be activated by DENV infection, which is consistent with the results of other studies. The JUN N-terminal kinase (JNK) pathway, mediated by the complement system and apoptosis, has broad-spectrum antiviral function against DENV-2, ZIKV and Chikungunya virus (CHIKV) in the salivary glands of *Ae. aegypti*, and co-silencing of the complement system and apoptosis can eliminate the antiviral effect of the JNK pathway (Chowdhury and Modahl, 2020). Martins Sde et al. (2012) reported that dengue virus infection of dendritic cells can induce apoptosis and impair their ability to present antigens to T cells, thereby promoting dengue pathogenesis.

DENV can infect many tissues including the spleen, lungs, liver and central nervous system (CNS) in human (Shresta et al., 2004; Bentes et al., 2021). Flaviviruses can invade the CNS through different mechanisms, including axonal transport in the peripheral nervous system, through the bloodstream pathway, by way of the infected monocytes and peripheral blood leukocytes, as well as *via* infection of blood-brain barrier endothelial cells (Bentes et al., 2021). Although the mechanism of infection of CNS is unclear, animal and cellular model studies suggest that these viruses share a common neuropathogenic pathway (Mustafá et al., 2019). The DEPs including A0A182G8T6 (Cytochrome b-c1 complex subunit 6), A0A023ENT8 (Putative short-chain alcohol dehydrogenase/3-hydroxyacyl-coa dehydrogenase), A0A023ETG9 are all related to the KEGG pathway of nervous system diseases, indicating that mosquito infection with DENV also causes neurological diseases. Among them, A0A023ETG9 belongs to microtubule (MT) protein, the development of the nervous system is highly dependent on the microtubule cytoskeleton, and mutations in microtubule protein can cause a series of neurological diseases (Tischfield et al., 2010). According to reports, depolymerization or stabilization of MTs can affect virus transport to the endoplasmic reticulum and virus production, and when MTs are disrupted, the number of released virus particles is significantly reduced (Buchwalter et al., 2021; Qi et al., 2022), the expression of A0A023ETG9 decreased in the virus-infected group, which indicates that MT is associated with DENV infection, and this could be a target to inhibit virus replication, but its principle and mechanism need to be further investigated.

PC protein, an important component of the periphagous membrane of insect intestines, is mosquito’s first natural barrier against the invasion of foreign microorganisms. Recent studies have shown the importance of reducing the penetration of insecticides into the stratum corneum as a mechanism of mosquito resistance (Saavedra-Rodriguez and Campbell, 2021). For example, thickening of the cuticle was associated with reduced permeability to pyrethroids in resistant mosquitoes in *Anopheles gambiae* (Yahouédo et al., 2017). The overexpression of cytochrome P450 (CYP) promotes the deposition of epidermal hydrocarbons in the epidermis of resistant mosquitoes, and penetration resistance occurs when large amounts of cuticular hydrocarbons form and deposit on the top of the cuticle to form a barrier, resulting in the slow absorption of pesticides into their bodies (Kasai et al., 2014; Bass and Jones, 2016). However, the relationship between PC and virus has rarely been reported. Previous study showed that AAEL011045, a pupal cuticle protein, can bind West Nile envelope protein (WNE) to inhibit the infection of West Nile virus *in vitro* and prevent the lethal WNV encephalitis in mice (Colpitts et al., 2011). Li and Denlinger (2009) found that the pupal cuticle protein is abundant in the early diapause stage of Culex mosquitoes to enhance the stress resistance of the cuticle. In this study, we found that A0A023EEV6, a PC protein could reduce the replication of DENV and interacted with DENV-E, indicating that PC protein might act at the step of viral entry, possibly by directly binding to the E protein on the virus surface. DENV-E is required for productive viral entry and initiation of infection. de Wispelaere et al. (2018) discovered compounds that inhibited DENV by binding DENV-E, and further demonstrated the inhibitory effect of these compounds on ZIKV, West Nile virus, and Japanese encephalitis virus. The changes in PC levels affect the composition and performance of the stratum corneum, while the stimulation by the external environment and the invasion of foreign pathogens can both increase PC levels in mosquitoes. We found that PC could inhibit the replication of DENV, and its potential application value, such as using PC protein molecules as antiviral target molecules to produce transgenic mosquitoes for prevention of dengue fever, and the mechanism of PC protein inhibiting DENV replication, all warrants further investigations.

The ubiquitin-proteasome system (UPS) regulates protein turnover in cells. UPS contains three basic principle components: Proteasome holoenzymes, multiple ubiquitin ligases, and several kinds of deubiquitinating enzymes (DUBs) (Bailey-Elkin et al., 2017). Ubiquitination/deubiquitination not only regulates protein turnover, but also controls protein function, protein localization, and protein-protein interactions. Many eukaryotic viruses have been observed to influence the host protein ubiquitination/deubiquitination machinery to facilitate their life cycle and aspects of their pathogenesis (Pumpuni et al., 1992; Troupin et al., 2016; Ali et al., 2017). Therefore, the ubiquitination/deubiquitination process is widely exploited by viruses (Nijman et al., 2005; Lindner, 2007; Isaacson and Ploegh, 2009; Lata et al., 2018). A0A023EVI6 belongs to a group of ubiquitin ligases, which can modify a series of proteins specifically, and the modified proteins can participate in regulating the immune response of the body, etc. Binding of the NS5 protein of DENV to STAT2 leads to ubiquitination of STAT2, resulting in a reduced type I interferon-mediated immune response, leading to a reduced ability to inhibit dengue virus replication (Mazzon et al., 2009). In DENV infection, knockdown of the E3 ubiquitinase SIAH1 gene reduces the ubiquitination level of MyD88 protein, thereby increasing the ability to inhibit DENV replication (Murphy-Schafer et al., 2020). AOAO23EXQ2 belongs to ubiquitin carboxy-terminal hydrolase (UCH), a member of the deubiquitination family. Recently, it was observed that the DENV NS5 alone could degrade a DUB protein, ubiquitin specific peptidase 42 (USP42) (Mishra et al., 2019) and DENV NS1 could degrades USP33 in human microglial cells (Mishra and Lahon, 2020). Ubiquitin carboxy-terminal hydrolase-L1 (UCH-L1) maintains cellular homeostasis under conditions of normal growth and oxidative stress (Shen et al., 2006), and the deletion of UCH-L1 impaired the homeostasis and metabolism of spermatogonial stem cells (SSCs) and affected their differentiation ability (Alpaugh and Voigt, 2021). However, there are few reports on the functional role of UCH in mosquitoes or the interaction between UCH and flaviviruses. In this study, we found that infection with DENV decreased the level of UCH, and DENV-E co-localized with UCH in BHK cells, indicating that UCH might interact with dengue virus, which requires further verification.

## Conclusion

In conclusion, iTRAQ proteomic analysis of the midgut of *Ae. albopictus* identified DENV-induced proteins. This study demonstrated that a putative pupal cuticle (PC) protein could inhibit the replication of DENV and interacted with DENV-E. The co-localization between ubiquitin carboxyl-terminal hydrolase (UCH) protein and DENV-E protein suggested that virus infection can reduce the level of this protein.

## Data availability statement

The original contributions presented in this study are included in the article/Supplementary material, further inquiries can be directed to the corresponding author.

## Author contributions

JTW and XLZ conceived, designed the experiments, analyzed the data, and wrote the manuscript. JTW, PYF, JQW, YW, and WHZ performed the experiments. XLZ revised the manuscript. All authors have read and approved the final manuscript.

## Footnotes

1
http://www.geneontology.org/

2
http://www.ncbi.nlm.nih.gov/COG/

3
http://www.genome.jp/kegg/

## Supplementary material

Supplementary Figure 1 Differentially expressed proteins (DEPs). **(A)** Statistics of up- and down-regulated DEPs in *Ae. albopictus*. **(B)** Each point in the graph represents a protein. Compared with the mock group, red indicates up-regulated, and blue indicates down-regulated.

Supplementary Figure 2 Repeatability analysis between three replicates of liquid chromatography with tandem mass spectrometry (LC-MS-MS) experiments in *Ae. albopictus* in response to DENV. The figure shows the correlation between three replicates of LC-MS/MS samples, the squares represent the correlation between the corresponding samples: The redder the color, the closer the correlation coefficient is to 1, and the larger the circle, the better the repeatability in-group sample. By contrast, the greener the color, the closer the correlation coefficient is to −1, and the larger the circle, the worse the repeatability of in-group samples. The correlation coefficients between the groups of the samples were all positive, showing a positive correlation and good repeatability.

Supplementary Figure 3 STRING analysis of protein-protein interactions between DEPs. Network of interactions of DEPs was performed using string v9.05. The proteins were analyzed at a confidence level 0.4. The interactions between DEPs were indicated by the connecting line. The thickness of the connecting line represented the strength of the associations.
