# Supplementary figures and images for: Isobaric tags for relative and absolute quantification-based proteomic analysis of host-pathogen protein interactions in the midgut of Aedes albopictus during dengue virus infection
Source: Front Microbiol. 2022 Sep 14;13:990978. doi: 10.3389/fmicb.2022.990978 (PMC9515977; doi:10.3389/fmicb.2022.990978)

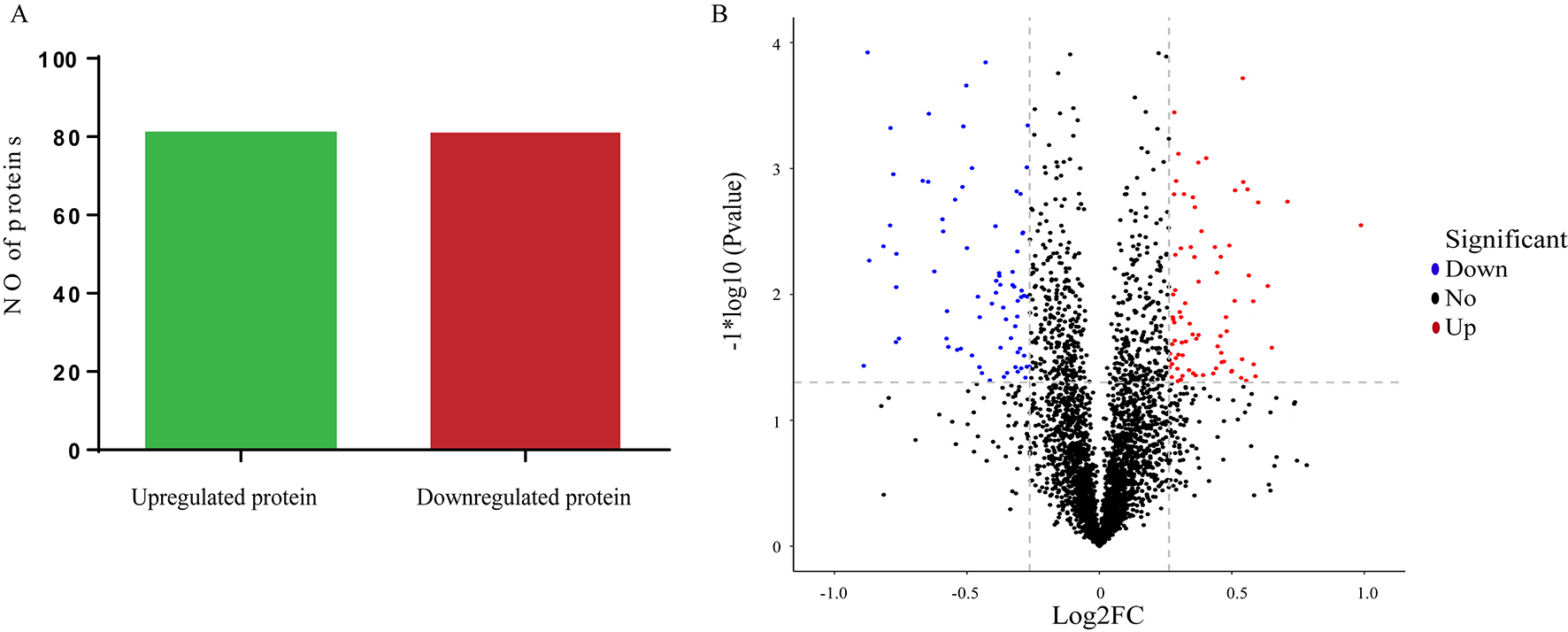

Supplement: Supplementary Figure 1 — Differentially expressed proteins (DEPs). (A) Statistics of up- and down-regulated DEPs in Ae. albopictus. (B) Each point in the graph represents a protein. Compared with the mock group, red indicates up-regulated, and blue indicates down-regulated. [file Image_1.TIF]

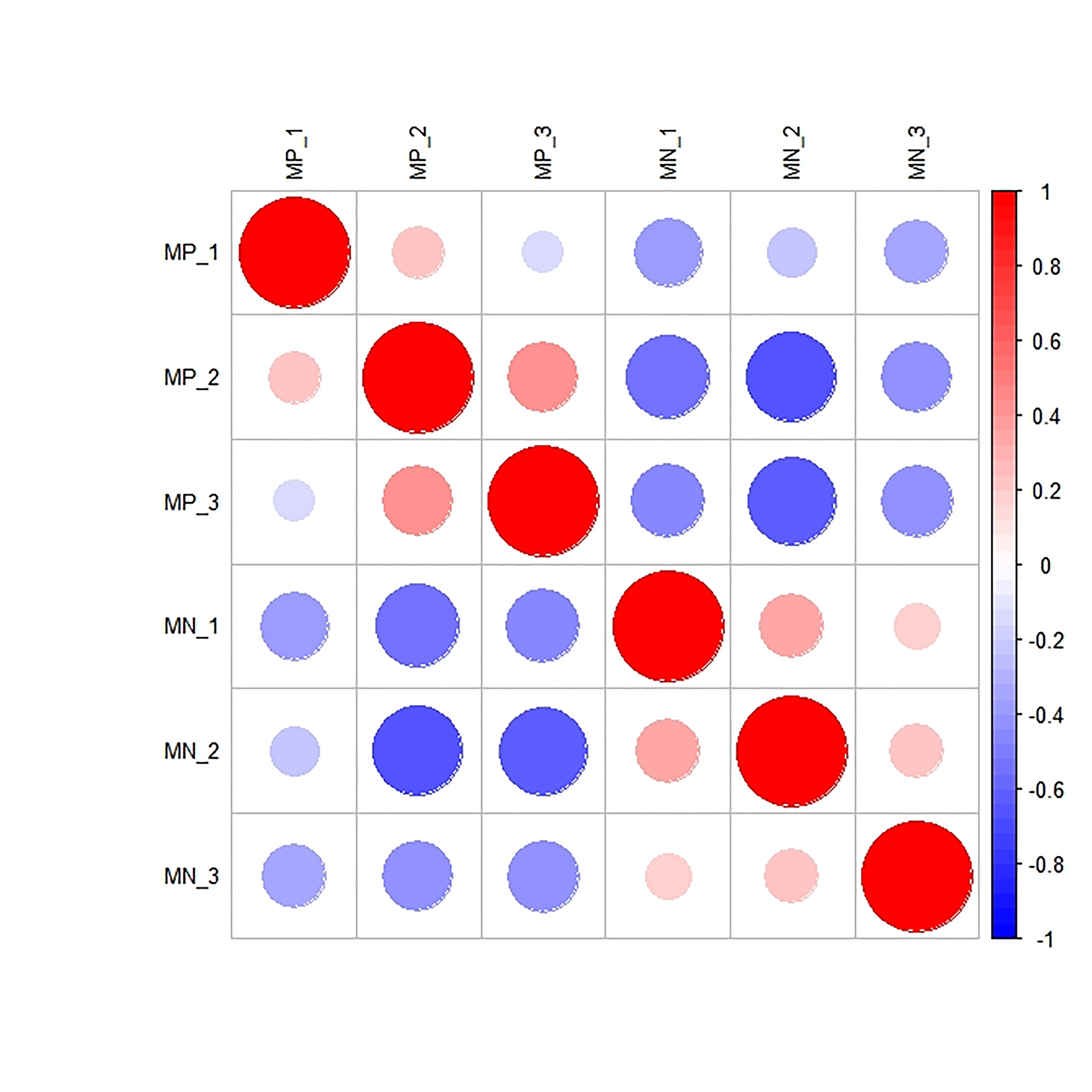

Supplement: Supplementary Figure 2 — Repeatability analysis between three replicates of liquid chromatography with tandem mass spectrometry (LC-MS-MS) experiments in Ae. albopictus in response to DENV. The figure shows the correlation between three replicates of LC-MS/MS samples, the squares represent the correlation between the corresponding samples: The redder the color, the closer the correlation coefficient is to 1, and the larger the circle, the better the repeatability in-group sample. By contrast, the greener the color, the closer the correlation coefficient is to −1, and the larger the circle, the worse the repeatability of in-group samples. The correlation coefficients between the groups of the samples were all positive, showing a positive correlation and good repeatability. [file Image_2.TIF]

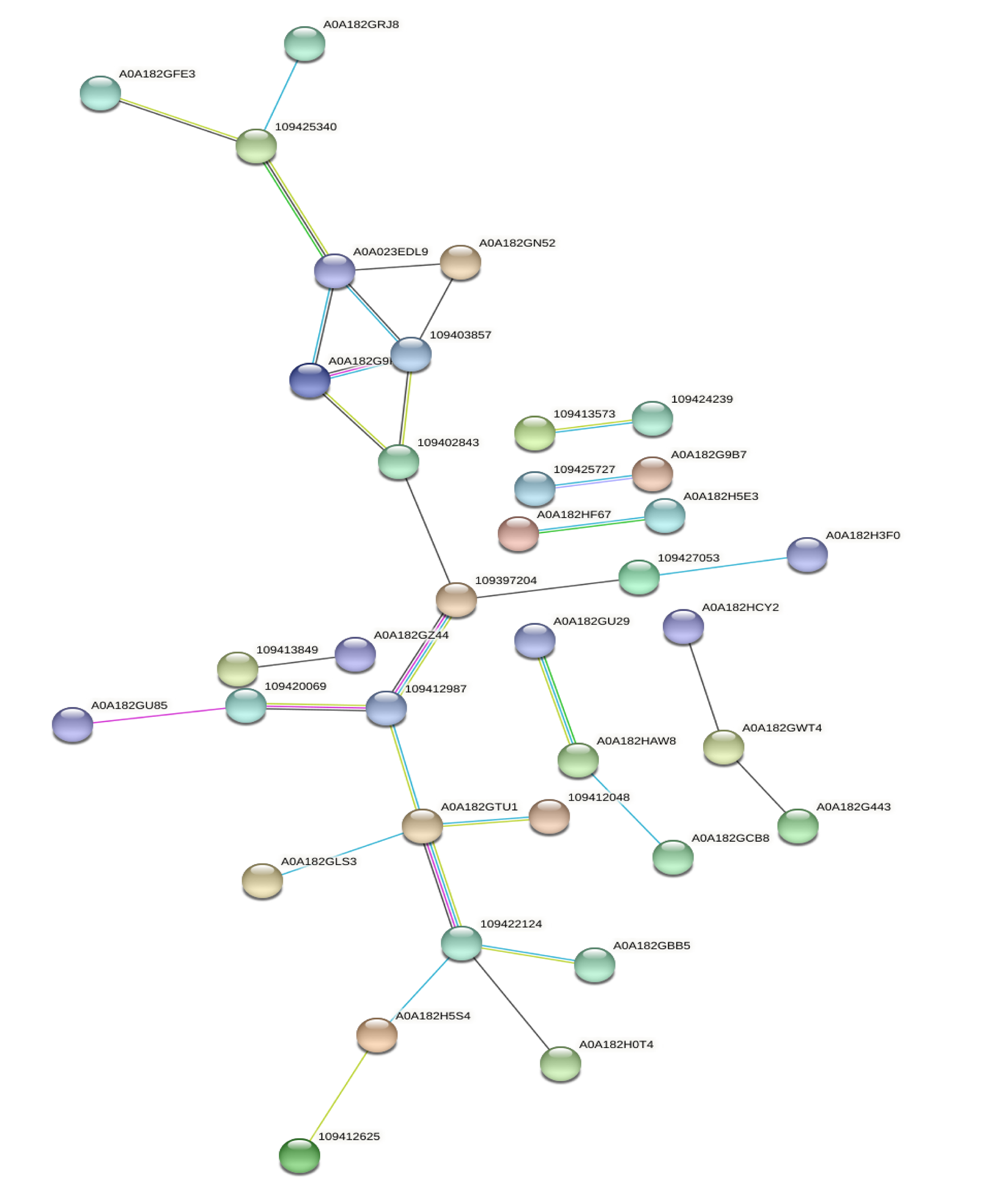

Supplement: Supplementary Figure 3 — STRING analysis of protein-protein interactions between DEPs. Network of interactions of DEPs was performed using string v9.05. The proteins were analyzed at a confidence level 0.4. The interactions between DEPs were indicated by the connecting line. The thickness of the connecting line represented the strength of the associations. [file Image_3.TIF]
