# Supplementary material for: Isobaric tags for relative and absolute quantification-based proteomic analysis of host-pathogen protein interactions in the midgut of Aedes albopictus during dengue virus infection
Source: Front Microbiol. 2022 Sep 14;13:990978. doi: 10.3389/fmicb.2022.990978 (PMC9515977; doi:10.3389/fmicb.2022.990978)
Supplement: Supplementary file 4 [file Table_1.DOCX]

**S1 Table Primer Information**

| Species | Gene | Primer name | Sequence 5'-3' | Fragment Length  (bp) |
| --- | --- | --- | --- | --- |
| *Aedes albopictus* | S7 | Rps7-F | CTGATGCGTTCGAGGGTCAA | 110 |
|  |  | Rps7-R | ACGCTCACCAATGAACACGA |  |
| Dengue virus 2 | POLY | DENV-3’utr-F | TCCCTTACAAATCGCAGCAAC | 127 |
|  |  | DENV-3’utr-R | TGGTCTTTCCCAGCGTCAAT |  |
| *Aedes albopictus* | PC | EEV6-F | ATGGTGAGCAAGGGCGAGGA | 345 |
|  |  | EEV6-R | CTACTTCTTCGGTATATGAG |  |
| *Aedes albopictus* | UCH | EXQ2-HINDⅢ | ATGGCTGATAGCGCTGGCGA | 981 |
|  |  | EXQ2-ECORⅠ | CTATTTCTGTGCTGTCACTTTGCC |  |
| *Aedes albopictus* | A0A023EDJ3 | RT-qPRC-F | GGTCAAATCCGCTCCGAAGT | 158 |
|  |  | RT-qPCR-R | GGGTGGTAACCGTTCTCGTC |  |
| *Aedes albopictus* | A0A023EEV6 | RT-qPCR-F | GTGAACTACGTCGCCGATGA | 134 |
|  |  | RT-qPCR-R | AAGTTGGGGTCATCTCGTGG |  |
| *Aedes albopictus* | A0A023EVI6 | RT-qPCR-F | CCGGTGGGTGTATGAGGAAG | 192 |
|  |  | RT-qPCR-R | ACAATCCGACATTCGGTGCT |  |
| *Aedes albopictus* | A0A1L2F0C2 | RT-qPCR-F | GTGGATCGTTATGCCTCGGA | 134 |
|  |  | RT-qPCR-R | GCAAGGTTTTCGCAGTCCAC |  |
| *Aedes albopictus* | A0A023EXQ2 | RT-qPRC-F | ACATTGCTCTGTCCTCACGG | 154 |
|  |  | RT-qPCR-R | TGCAAATGTGGCACGGAAAG |  |
| *Aedes albopictus* | A0A023EME7 | RT-qPCR-F | TTGTGCGAGATGTATGCCCC | 198 |
|  |  | RT-qPCR-R | GAAGGCAGCCAAAGCAATGT |  |
| *Aedes albopictus* | A0A023EQS8 | RT-qPCR-F | TCCCGAAATCGGTGACCAAG | 151 |
|  |  | RT-qPCR-R | GGATGATGTGGATGGCCGAA |  |
| *Aedes albopictus* | A0A023ETG9 | RT-qPCR-F | GAGGGATGCGATTGCCTTCA | 148 |
|  |  | RT-qPCR-R | TTGGCGATGGAACAACGGA |  |
