# Supplementary material for: Isobaric tags for relative and absolute quantification-based proteomic analysis of host-pathogen protein interactions in the midgut of Aedes albopictus during dengue virus infection
Source: Front Microbiol. 2022 Sep 14;13:990978. doi: 10.3389/fmicb.2022.990978 (PMC9515977; doi:10.3389/fmicb.2022.990978)
Supplement: Supplementary file 5 [file Table_2.DOCX]

**S2 Table DENV-Ⅱ infection rates of *Aedes albopictus* in the experimental group**

| Group number | Infection rate | Viral load (*copies*) |
| --- | --- | --- |
| 1 | 80% | 4.88*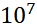 |
| 2 | 80% | 3.80*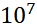 |
| 3 | 90% | 4.86*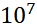 |
