# Supplementary material for: Isobaric tags for relative and absolute quantification-based proteomic analysis of host-pathogen protein interactions in the midgut of Aedes albopictus during dengue virus infection
Source: Front Microbiol. 2022 Sep 14;13:990978. doi: 10.3389/fmicb.2022.990978 (PMC9515977; doi:10.3389/fmicb.2022.990978)
Supplement: Supplementary file 6 [file Table_3.DOCX]

**S3 Table List of differentially expressed proteins in the midgut**

| Protein ID | Protein name | Infected:Mock | P value |
| --- | --- | --- | --- |
| **UP-regulation** | | | |
| Q5MIP7 | ATPase protein 9 | 1.37578125 | 0.029054345 |
| Q5MIP9 | ATP synthase-coupling factor 6, mitochondrial | 1.286200378 | 0.04383096 |
| A0A023EBL5 | Protein MGR2 homolog | 1.254101488 | 0.023691015 |
| A0A023EVC6 | Putative relative of woc | 1.37366548 | 0.005022471 |
| A0A023EDJ3 | Putative acp65aa | 1.497510373 | 0.035929371 |
| A0A023EFJ1 | Putative pretaporter | 1.295790671 | 0.000894568 |
| A0A023EQJ3 | Putative mrna export protein | 1.20226774 | 0.037897425 |
| A0A023EDU6 | Putative acp65aa | 1.449159327 | 0.045919007 |
| A0A023EVZ4 | Alpha-amylase | 1.222631773 | 0.001257851 |
| A0A023ETT2 | Putative adenylate cyclase terminal-differentiation specific | 1.35711501 | 0.038692592 |
| A0A023EP55 | Glutaminyl-peptide cyclotransferase | 1.277171869 | 0.001690429 |
| A0A023ET05 | High temperature requirement protein A2 | 1.234426834 | 0.013790137 |
| A0A023ETM4 | Putative pppde peptidase domain protein | 1.238095238 | 0.004303646 |
| A0A1W7R535 | BTB domain-containing protein | 1.414518048 | 0.040312236 |
| A0A023EXY8 | SOSS complex subunit A homolog | 1.220588235 | 0.004844535 |
| A0A023EWM5 | Rab proteins geranylgeranyltransferase component | 1.213030747  3 | 0.015765991 |
| A0A023EJ67 | Putative ca2+ sensor ef-hand superfamily | 1.636323851 | 0.00183063 |
| A0A1W7R6T2 | Putative salivary vascular endothelial growth factor | 1.266915888 | 0.017046994 |
| A0A023ESA9 | Putative e3 ubiquitin-protein ligase bre1 | 1.478600823 | 0.007061756 |
| A0A023EUN6 | Putative mitotic protein phosphatase 1 regulator | 1.216660577 | 0.016698235 |
| A0A1W7R6G5 | Reverse transcriptase | 1.424646465 | 0.01122564 |
| A0A023EE92 | Putative cytochrome oxidase complex assembly protein 1 | 1.57026214 | 0.026496493 |
| A0A023EHI4 | Putative glutathione s-transferase | 1.283885542 | 0.002028358 |
| A0A1W7R7A9 | Putative serine protease inhibitor serpin | 1.412673267 | 0.041094644 |
| A0A023EPM2 | Putative n-methyl-d-aspartate receptor glutamate-binding subunit | 1.353284104 | 0.004201959 |
| A0A023EVI6 | Putative e3 ubiquitin ligase cullin 2 component | 1.468224682 | 0.048408718 |
| A0A1L2F0C2 | Caspase 7 | 1.23811279 | 0.047555679 |
| A0A023EPK3 | Putative secreted protein | 1.306192661 | 0.003148981 |
| A0A023EM89 | Putative trna-binding protein | 1.473512632 | 0.00146336 |
| A0A023ECD8 | Small nuclear ribonucleoprotein E | 1.264761905 | 0.039927169 |
| A0A023ETS0 | Putative beta-catenin-tcf/lef signaling pathway component drctnnb1a | 1.278404816 | 0.042290953 |
| A0A023EEV6 | Putative pupal cuticle protein | 1.201019665 | 0.033320838 |
| A0A023EBH5 | Putative mitochondrial cytochrome c oxidase assembly protein/cu2+ chaperone cox17 | 1.395422257 | 0.019571753 |
| A0A023EG78 | Putative secreted salivary protein | 1.515468227 | 0.001856506 |
| A0A023ETC3 | Putative goliath e3 ubiquitin ligase | 1.208378871 | 0.045313483 |
| A0A023EIG0 | Putative secreted protein | 1.457304735 | 0.001280365 |
| A0A182H0T4 | Pre-mRNA-splicing factor 38 | 1.215829974 | 0.001601071 |
| A0A182GLS3 | Non-specific serine/threonine protein kinase | 1.226449945 | 0.038905325 |
| A0A182G3B0 | Prolyl-tRNA synthetase | 1.452545825 | 0.032730971 |
| A0A182GTT5 | Senescence domain-containing protein | 1.227941176 | 0.048850461 |
| A0A182GLV5 | Midasin | 1.3475205 | 0.042441926 |
| A0A182GRP5 | ACB domain-containing protein | 1.221812822 | 0.03193233 |
| A0A182G8T6 | Cytochrome b-c1 complex subunit 6 | 1.504930156 | 0.044630989 |
| A0A182H9X9 | LysM domain-containing protein | 1.282897232 | 0.005047246 |
| A0A182GN52 | Complex I-9kD | 1.311902028 | 0.043719934 |
| A0A182GL39 | Tyrosinase_Cu-bd domain-containing protein | 1.296619825 | 0.00790261 |
| A0A182H5U7 | Rab-GAP TBC domain-containing protein | 1.252059925 | 0.011750201 |
| A0A182GZ75 | RING-type domain-containing protein | 1.373328088 | 0.021376057 |
| A0A182GHH5 | Amidase domain-containing protein | 1.243433222 | 0.030394098 |
| A0A182H5S4 | NR LBD domain-containing protein | 1.983134921 | 0.002823664 |
| A0A182H7G3 | CBM39 domain-containing protein | 1.362821512 | 0.025861408 |
| A0A182GVW2 | Mediator complex subunit 23 | 1.553556485 | 0.008561935 |
| A0A182GHV1 | Geranylgeranyl transferase type-2 subunit beta | 1.243122677 | 0.044318511 |
| A0A182GEA9 | Microsomal prostaglandin E synthase 2 | 1.209541985 | 0.035629634 |
| A0A023EFI2 | Putative 14.5 kDa salivary peptide | 1.322680015 | 0.000827323 |
| A0A182G104 | Galactosylgalactosylxylosylprotein 3-beta-glucuronosyltransferase | 1.270433145 | 0.004208325 |
| A0A182GQ65 | Phosphatidate cytidylyltransferase, mitochondrial | 1.385623511 | 0.034119796 |
| A0A182HDT2 | Uncharacterized protein | 1.211609111 | 0.015148172 |
| A0A182GF96 | Uncharacterized protein | 1.229809104 | 0.030077634 |
| A0A023EML1 | Uncharacterized protein | 1.203062341 | 0.029519168 |
| A0A023EJQ7 | Uncharacterized protein | 1.209872029 | 0.024843365 |
| A0A023EUY2 | Uncharacterized protein | 1.426741393 | 0.001487616 |
| A0A182GFE3 | Uncharacterized protein | 1.238182484 | 0.015117489 |
| A0A182GU29 | Uncharacterized protein | 1.219646799 | 0.009241726 |
| A0A182H4R4 | Uncharacterized protein | 1.393081761 | 0.015153979 |
| A0A182GBC7 | Uncharacterized protein | 1.360031104 | 0.006705082 |
| A0A182GJD2 | Uncharacterized protein | 1.216849817 | 0.000357364 |
| A0A182GRJ8 | Uncharacterized protein | 1.230022405 | 0.000763433 |
| A0A182GHA1 | Uncharacterized protein | 1.455128205 | 0.000191431 |
| A0A182GMH1 | Uncharacterized protein | 1.404998017 | 0.004088488 |
| A0A182H6V4 | Uncharacterized protein | 2.136196962 | 0.012321313 |
| A0A182H3X6 | Uncharacterized protein | 1.202112163 | 0.033300724 |
| A0A182HE99 | Uncharacterized protein | 1.248053393 | 0.001595492 |
| A0A182H2I3 | Uncharacterized protein | 1.49589491 | 0.011314979 |
| A0A182GZ85 | Uncharacterized protein | 1.289015152 | 0.022512129 |
| A0A182GWT4 | Uncharacterized protein | 1.296239447 | 0.02097716 |
| A0A182GZ44 | Uncharacterized protein | 1.276547842 | 0.02074083 |
| A0A182H670 | Uncharacterized protein | 1.240999265 | 0.02410913 |
| A0A182GEJ6 | Uncharacterized protein | 1.377543036 | 0.034574265 |
| A0A182H1M3 | Uncharacterized protein | 1.212852897 | 0.010015779 |
| A0A182GDK6 | Uncharacterized protein | 1.218201754 | 0.023281219 |
| **Down-regulation** | | | |
| Q5MIU5 | Putative 56 kDa salivary secreted protein | 0.547581903 | 0.005378077 |
| Q5MIR1 | Putative 60s ribosomal protein l24 | 0.806682578 | 0.014994408 |
| Q5MIU7 | Putative 56 kDa family 56k-2 salivary secreted protein | 0.805455635 | 0.001518772 |
| A0A023EHD1 | Putative ubiquitin protein ligase glossina morsitans morsitans | 0.802343046 | 0.017881165 |
| A0A023EFW0 | Putative flightin | 0.579002902 | 0.000475903 |
| A0A023ETG9 | Tubulin beta chain | 0.769411765 | 0.006763694 |
| A0A182HFF4 | GH16 domain-containing protein | 0.58315565 | 0.001108855 |
| A0A023EQX2 | Ubiquitin carboxyl-terminal hydrolase | 0.829290479 | 0.000454201 |
| A0A023EIP0 | Putative microtubule associated complex | 0.779232371 | 0.045220653 |
| A0A1W7R4P7 | N-acetyltransferase domain-containing protein 1 | 0.699007092 | 0.001398766 |
| A0A023EJ07 | Putative chromobox protein 1 | 0.816221142 | 0.009315538 |
| A0A023ER22 | Putative beta-glucosidase lactase phlorizinhydrolase | 0.751096812 | 0.048224832 |
| A0A023EF21 | 40S ribosomal protein S26 | 0.754954955 | 0.011849641 |
| A0A023EP68 | Putative microtubule-associated protein | 0.587890104 | 0.004756573 |
| A0A023ESR7 | Putative sirtuin 5 | 0.806896552 | 0.00455319 |
| A0A023EXR0 | Aconitate hydratase, mitochondrial | 0.827156744 | 0.037503115 |
| A0A023EHP6 | Putative secreted protein | 0.813503867 | 0.001592114 |
| A0A023EJG8 | Putative phosphatidylethanolamine-binding protein | 0.783239562 | 0.015748471 |
| A0A023EQS8 | Putative aldo/keto reductase family | 0.664630761 | 0.003153525 |
| A0A1W7R8G2 | Glyoxalase domain-containing protein 4 | 0.829631883 | 6.19E-05 |
| A0A023EME7 | Putative glutathione s-transferase | 0.762886598 | 0.009686152 |
| A0A023EMF0 | S-formylglutathione hydrolase | 0.673651858 | 0.026080043 |
| A0A023EIG7 | Putative dehydrogenase | 0.629760609 | 0.001250757 |
| A0A023EM09 | Proteasome subunit beta | 0.802897676 | 0.037862382 |
| A0A023EQ19 | Putative metalloexopeptidase | 0.696047768 | 0.026983066 |
| A0A023EK84 | Tetraspanin | 0.821471653 | 0.010234633 |
| A0A023ERE4 | Putative inositol monophosphatase | 0.815151515 | 0.038607385 |
| A0A023EDH9 | Putative sterol carrier protein-2 like-3 variant 1 | 0.772248244 | 0.026468132 |
| A0A023ENT8 | Putative short-chain alcohol dehydrogenase/3-hydroxyacyl-coa dehydrogenase | 0.807669082 | 0.04106419 |
| A0A023ERR0 | J domain-containing protein | 0.812652068 | 0.026917478 |
| A0A023EWE5 | Putative hiv-1 vpr-binding protein | 0.777810477 | 0.012737957 |
| A0A023EMX8 | Putative elongation factor 1 beta/delta chain | 0.818072289 | 0.003277402 |
| A0A182HC04 | Dipeptidyl peptidase 3 | 0.671244395 | 0.013590491 |
| A0A023ELT0 | Putative aprataxin and pnk-like factor | 0.670305066 | 0.022373641 |
| A0A1W7R8W4 | Putative chain a | 0.771748747 | 0.008372676 |
| A0A023EU85 | Putative glucose dehydrogenase/choline dehydrogenase/mandelonitrile lyase gmc oxidoreductase family | 0.68956743 | 0.027571797 |
| A0A023EFJ4 | Protein serine/threonine kinase | 0.796832985 | 0.00841496 |
| A0A182HF67 | Tox-SGS domain-containing protein | 0.431914894 | 0.007514862 |
| A0A182G197 | Component of oligomeric Golgi complex 7 | 0.821493625 | 0.030595737 |
| A0A023ECJ5 | Putative secreted protein | 0.807669263 | 0.028781404 |
| A0A182G4T7 | Ig-like domain-containing protein | 0.707056508 | 0.004281389 |
| A0A182H5E3 | FHA domain-containing protein | 0.78567217 | 0.042000775 |
| A0A182G9B8 | SHSP domain-containing protein | 0.762131587 | 0.00287509 |
| A0A182H411 | PUA domain-containing protein | 0.735353535 | 0.042127306 |
| A0A182GMZ4 | Exonuclease domain-containing protein | 0.807575758 | 0.011240717 |
| A0A182H4V3 | zf-AD domain-containing protein | 0.476816266 | 0.000371018 |
| A0A182GHS7 | G domain-containing protein | 0.82413273 | 0.045772766 |
| A0A023EFS5 | 40S ribosomal protein S30 | 0.591727102 | 0.022389136 |
| A0A182H6Q5 | N-acetyltransferase domain-containing protein | 0.731239092 | 0.015164547 |
| A0A182H3F0 | Sulfite oxidase | 0.705832148 | 0.000219203 |
| A0A182G9B7 | SHSP domain-containing protein | 0.818923821 | 0.003216656 |
| A0A023EDH3 | Ubiquitin-fold modifier 1 | 0.381007128 | 0.001530849 |
| A0A182GEK2 | U-box domain-containing protein | 0.54379186 | 2.34E-05 |
| A0A023END4 | Triosephosphate isomerase | 0.663219676 | 0.002527483 |
| A0A182GQW0 | Homeobox domain-containing protein | 0.796645702 | 0.00661849 |
| A0A182GTU1 | NEDD8 | 0.568235603 | 9.84E-06 |
| A0A182G7B7 | UDP-glucuronosyltransferase | 0.827217125 | 0.000976182 |
| A0A182GB27 | Protein Wnt | 0.545290323 | 0.000119627 |
| A0A023EDL9 | V-type proton ATPase subunit F | 0.800178678 | 0.008675531 |
| A0A182GYL9 | Uncharacterized protein | 0.74268328 | 0.000143056 |
| A0A182H4D0 | Uncharacterized protein | 0.539825283 | 0.036885995 |
| A0A182GQA6 | Uncharacterized protein | 0.833082254 | 0.037354005 |
| A0A182G3U0 | Uncharacterized protein | 0.815204327 | 0.010514795 |
| A0A182HE83 | Uncharacterized protein | 0.578354298 | 0.002826836 |
| A0A182GF33 | Uncharacterized protein | 0.649315068 | 0.006561153 |
| A0A182GTF4 | Uncharacterized protein | 0.827943868 | 0.010421007 |
| A0A182GKN6 | Uncharacterized protein | 0.769750367 | 0.007110846 |
| A0A182H3T9 | Uncharacterized protein | 0.640273973 | 0.000367455 |
| A0A182GD62 | Uncharacterized protein | 0.730724638 | 0.037753846 |
| A0A182HAW8 | Uncharacterized protein | 0.727768216 | 0.01043208 |
| A0A182HCY2 | Uncharacterized protein | 0.716685649 | 0.030526589 |
| A0A182GIS6 | Uncharacterized protein | 0.716700086 | 0.000990245 |
| A0A182G443 | Uncharacterized protein | 0.763466042 | 0.007802988 |
| A0A182HE46 | Uncharacterized protein | 0.380697674 | 0.000758859 |
| A0A182GCB8 | Uncharacterized protein | 0.793366598 | 0.022278844 |
| A0A182G9R8 | Uncharacterized protein | 0.568409931 | 0.00414523 |
| A0A182GU85 | Uncharacterized protein | 0.700595407 | 0.000463296 |
| A0A182H2T8 | Uncharacterized protein | 0.587587319 | 0.008769534 |
| A0A182GGB4 | Uncharacterized protein | 0.587263523 | 0.023962308 |
| A0A182GBB5 | Uncharacterized protein | 0.685569905 | 0.001767177 |
| A0A182G7E8 | Uncharacterized protein | 0.638926909 | 0.001274057 |
